# Fertility preservation in female cancer patients in Brazil: perceptions and attitudes of infertility specialists

**DOI:** 10.61622/rbgo/2024rbgo25

**Published:** 2024-04-09

**Authors:** Renata Lack Ranniger, Rívia Mara Lamaita, Bárbara Flecha D’Abreu, Mariana Rodrigues Tolentino, Eduardo Batista Cândido, Warne Pedro Andrade, Angélica Nogueira-Rodrigues, Agnaldo Lopes Silva-Filho

**Affiliations:** 1 Universidade Estadual Paulista "Júlio de Mesquita Filho" Faculdade de Medicina de Botucatu Department of Gynecology Botucatu SP Brazil Department of Gynecology, Obstetrics and Mastology, Faculdade de Medicina de Botucatu, Universidade Estadual Paulista "Júlio de Mesquita Filho", Botucatu, SP, Brazil.; 2 Universidade Federal de Minas Gerais Department of Gynecology and Obstetrics Belo Horizonte MG Brazil Department of Gynecology and Obstetrics, Universidade Federal de Minas Gerais, Belo Horizonte, MG, Brazil.; 3 Mater Dei Department of Assisted Reproduction Belo Horizonte MG Brazil Department of Assisted Reproduction, Mater Dei, Belo Horizonte, MG, Brazil.; 4 Universidade Federal de Minas Gerais Department of Internal Medicine Belo Horizonte MG Brazil Department of Internal Medicine, Universidade Federal de Minas Gerais, Belo Horizonte, MG, Brazil.

**Keywords:** Fertility preservation, Reproduction, Fertility, Neoplasms, Oocyte retrieval, Health knowledge, attitudes, practice, Gynecologists, Oncologists, Surveys and questionnaires

## Abstract

**Objective:**

Fertility preservation is a priority in oncology for female cancer patients. However, there is a lack of communication between infertility specialists and oncologists. This study aimed to evaluate infertility specialists’ perceptions and experiences regarding fertility preservation.

**Methods:**

Conduct an online survey to profile infertility specialists. Participants were infertility affiliated with the Brazilian Federation of Gynecology and Obstetrics Associations (FEBRASGO). The specialists received an online survey, which response rate were 30.9%, most of whom were in southern and southeastern. The survey consisted on 14 questions about the infertility specialists’ location, techniques in clinical practice, treatment successful rate, patients idea, etc.

**Results:**

The average experience in human reproduction were 15.5 ± 10.2 years (mean ± standard deviation, range 1-40). Among reproductive-aged female cancer patients recommended for fertility preservation, 60.3 ± 28.8% (range 10-100%) underwent preservation procedures. Main barriers were cost (41%), oncologists’ knowledge or acceptance (35%) and accessibility (9%). Most infertility specialists (58%) considered 40 years the limit for fertility preservation. Leukemia, lymphoma, breast and ovarian cancers were prioritized for fertility preservation, while lung, thyroid, gastric, and brain cancers were less relevant.

**Conclusion:**

This is the first Brazilian study about infertility specialists’ perceptions on oncology patients access to fertility preservation. These patients primarily receive treatment in the public health system, while infertility specialists mainly work in the private healthcare. This healthcare mode is currently fragmented, but integrating these experts is enhancing patient access to fertility preservation. Studies on this topic are still warranted.

## Introduction

Fertility preservation is the practice of helping patients who are concerned about future infertility to preserve their chances of future reproduction. The impact of cancer treatment on fertility depends on the type and stage of cancer, the type of treatment, and the age at the time of treatment. Patients, who will undergo treatment that may affect their fertility, such as surgery, radiotherapy, chemotherapy, or hormone therapy, are candidates for fertility preservation techniques. Currently, fertility preservation is possible via freezing of embryos, eggs, and ovarian tissue as well as ovarian transposition (oophoropexy). The use of analogs of the gonadotropin-releasing hormone (GNRH) for ovarian suppression and to protect the ovaries from chemotherapy-induced damage still needs further clarification. In Brazil, there is no adequate financial support for patients who will undergo fertility preservation techniques in public health institutions. Therefore, most patients will undergo treatment in the private healthcare sector.

In Brazil, an estimate of 316,280 new cancer cases occurs in women annually. Excluding non-melanoma skin cancers, the malignant neoplasms that affect Brazilian women most frequently are breast cancers, followed by colorectal, cervical, lung, and thyroid cancers.^(1)^ In the United States, the overall mortality rate of different cancer types has decreased, and the 5-year survival rate has increased. These findings suggest that cancer patients have a longer life expectancy.^(2)^

The main goal of cancer treatment is to cure the disease and increase the survival of the patient. In recent years, the number of cancer survivors has significantly increased, and their quality of life has been a concern during cancer treatment.^(3,4)^ Loss of gonadal function by cancer treatment compromised the women´s reproductive future. Additionally, it is associated with sexual dysfunction and damage to the skeletal, cardiovascular, and neurological systems. Therefore, preserving ovarian function is an important determinant of the quality of life in female cancer patients.^(4,5)^

Comprehensive care for cancer patients includes counseling about possible threats to fertility in an agile, efficient, and multidisciplinary decision-making process.^(4,5)^ It is essential to refer women of reproductive age, who are proposed for oncologic treatment, to a reproductive medicine specialist. In this regard, an approach to counseling, risk-and-benefit evaluation of available therapies, and possible fertility preservation options, either by freezing of oocytes, embryos, or ovarian tissue, should be discussed.^(5,6)^

Although reproductive planning is a part of the healthcare model in oncological treatment, fertility preservation is not always routinely considered in the patient´s care plan. Costs, access to specialized services in assisted reproduction, the perceptions of this need by clinical oncologists, and the time required to institute the appropriate methods for oncofertility are factors that may limit the offer of fertility preservation to female cancer patients.^(6-8)^

This study aimed to evaluate the perceptions of and attitudes of infertility specialists about fertility preservation among female cancer patients. The results of this study may help define the strategies to integrate oncofertility into the healthcare model for female cancer patients in Brazil.

## Methods

After receiving the approval from the institutional review board of Brazilian Federation of Associations of Gynecology and Obstetrics (FEBRASGO), a full mailing list of infertility specialists was obtained. A questionnaire was developed to collect data about perceptions and attitudes of these infertility specialists from all five Brazilian regions (southeast, south, midwest, north, and northeast). The survey included 14 questions and took approximately 15 minutes to complete. This survey was submitted to the Commission of Ethics and Research of MaterDei Hospital. All participants received and signed a contract of free and informed agreement. The researchers committed to ensure the confidentiality of the collected data and the anonymity of the interviewees. All collected data were stored by FEBRASGO and shared with the participants. The questionnaire was made available through a free survey website (SurveyMonkey, San Mateo, CA, USA). E-mails were sent to all 323 FEBRASGO infertility specialists on April 19, 2021. The answers were received via the internet until May 20, 2021. Specialists who did not want to participate or those who provided incomplete responses (responded to less than 70% of the questions) were excluded. As illustrated in chart 1, the 14 questions addressed important issues about oncofertility, such as the number of technical procedures performed by an infertility specialist in order to preserve the patient's fertility in a future pregnancy. Depending on the fertility technique, one procedure may differ from the other. Survey data also included participants’ demographic and professional characteristics, including their current practice setting, experience with fertility, and perceptions regarding different cancers and barriers to oncofertility preservation.

**Chart 1:** Survey questions and the percentages of answers

Questions	Answered questions (%)
In which state do you work as a doctor?	100
How long have you been working in human reproduction (years)?	100
In your current practice, do you work in the private, SUS, or mixed network?	100
How many infertility procedures do you attend per year with a social indication and how many with an oncological indication?	96
Among the oncofertility techniques below, list in order of frequency the most common in your clinical practice.	89
Which age limit would you recommend for preserving fertility in a woman with cancer?	100
Among the malignant neoplasms below, list in order of frequency the most common in your clinical practice with an indication of fertility preservation.	92
Among the cancer patients seen and with an indication of fertility preservation, what was the percentage of patients who chose to undergo the procedure?	93
Among the cancer patients who underwent fertility preservation, what was the percentage of pregnancy?	42
Among the cancer patients who underwent fertility preservation, what was the percentage of live births?	49
I consider that fertility preservation is safe and does not change the oncological prognosis.	100
Should the possibility of fertility preservation be offered to all female cancer patients of reproductive age?	100
In your opinion, what are the main barriers to fertility preservation in women with cancer? List in order of importance.	96
In the referred cases, was there a multidisciplinary meeting with a specialist in Clinical Oncology for the decision-making process?	100

Survey data were analyzed using frequency distributions tests for various types of variables. Categorical variables were compared with the Chi-squared test and the Fisher exact test. Numerical variables were used compared with the Mann-Whitney test and the Kruskal-Wallis test. Survey results with answers for less than 70% of all questions were discarded. All unknown or missing responses were removed from the analysis. All statistical analyses were performed using IBM SPSS Statistics for Windows, Version 22.0 (IBM, Armonk, NY, USA). Statistical significance was set at p < 0.05.

## Results

**Figure 1:**
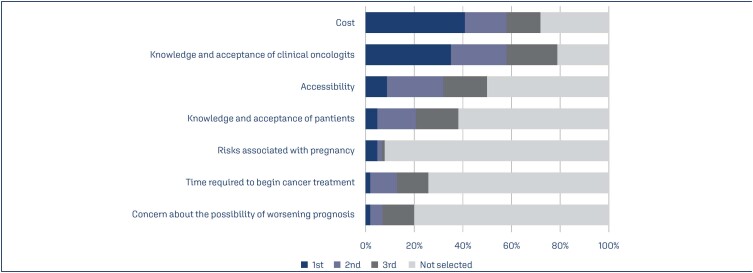
Perceived main barriers to fertility preservation in women with cancer

A fully completed questionnaire was returned by 30.9% FEBRASGO infertility specialists (100/323). All the received answers were included in the current analysis. Participants came from all Brazilian regions, with 64% from the southeast, 16% from the south, 10% from the midwest, 6% from the northeast, and 4% from the north. The participants’ average experience in human reproduction was 15.5 ± 10.2 years (mean ± standard deviation, range 1-40 years). Most specialists practiced only in the private health sector (52%), 45% worked in both private and public healthcare sectors, and 3% only worked in the public healthcare sector. The annual average number of non-oncological fertility preservation procedures of the participating infertility specialists was 29 ± 35.2 treatments (range 10-200 treatments). The annual average number of oncofertility preservation procedures was 6.3 ±9.4 treatments (range 1-80 treatments). Among female cancer patients with reproductive age and indicated fertility preservation, 60.3 ± 28.8% performed the procedure (range 10-100%). As illustrated in figure 1, the three main perceived barriers to fertility preservation in female cancer patients were cost (41%), knowledge and acceptance of clinical oncologists (35%), and service accessibility (9%).

**Figure 2:**
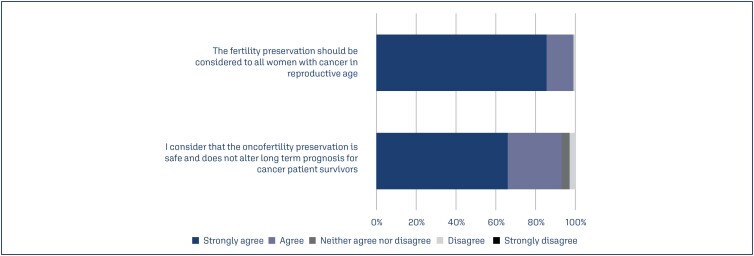
Perceptions of infertile specialists regarding the indications and safety of fertility preservation in women with cancer

As illustrated in figure 2, 99% of the respondents strongly agreed that fertility preservation should be considered in female cancer patients for their reproductive health. The statement that oncofertility preservation is safe and does not alter long-term cancer prognosis showed a strong agreement (93%) among infertility specialists.

**Figure 3:**
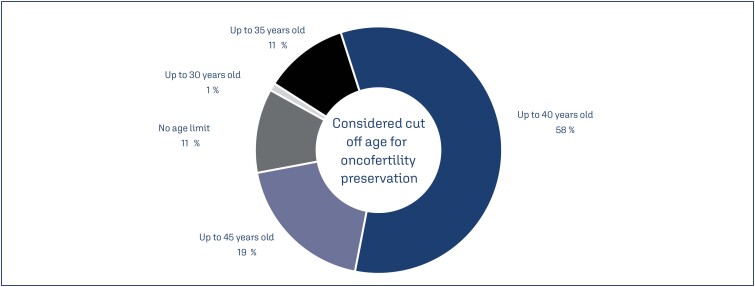
The age threshold for fertility preservation a woman with cancer that was considered by the survey participants

While most infertility specialists (58%) considered age above 40 years as the limit for fertility preservation indication in female patients with cancer (Figure 3), 11% of the respondents considered that there was no age limit for this procedure.

**Figure 4:**
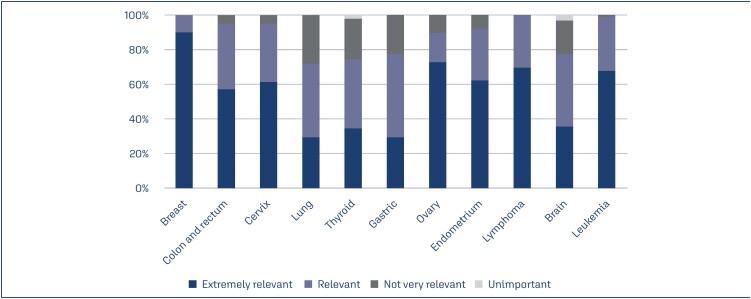
The relevance of fertility preservation for women among different malignancies

As illustrated in figure 4, breast and ovarian cancers, leukemia, and lymphoma were the most relevant malignant neoplasms for fertility preservation. In contrast, lung, thyroid, gastric, and brain cancers were less relevant for oncofertility preservation purposes. A multidisciplinary meeting with a clinical oncologist for the decision-making process in fertility preservation was conducted in 70% cases.

**Figure 5:**
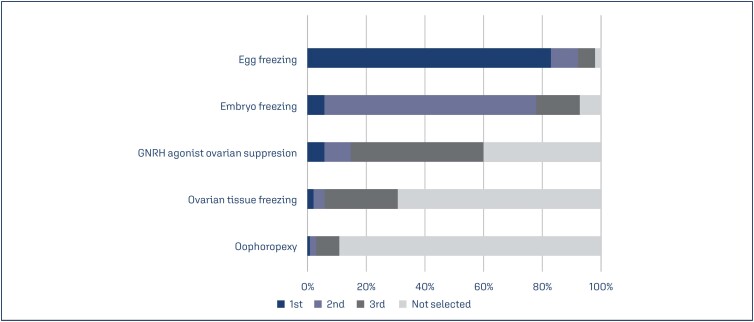
The most used techniques for fertility preservation in women with cancer

Egg freezing (81%) was the most commonly used technique for fertility preservation in women with cancer, followed by embryo freezing (6%), ovarian-suppression GNRH agonist (6%), and ovarian tissue freezing (2%) (Figure 5). Regarding fertility outcomes, most experts reported that they lost follow-up after the initiation of fertility preservation strategies. The specialists did not know the resulting pregnancy (50%) and live birth (86%) rates.

## Discussion

An estimate of 10% cancer cases affect women under 45 years of age with possible reproductive desire, Among these female cancer patients, approximately 83% survived the disease.^(9,10)^ Therefore, providing fertility preservation and its counseling could improve the quality of life of thousands of patients worldwide. A small proportion of cancer patients of reproductive age are referred to oncofertility specialists around the world.^(6)^ In this regard, a study of 167 Japanese adolescents and young adults showed that only 48% of the patients with non-breast and non-hematological malignancies received information about oncofertility.^(11)^ Oncofertility data from Brazil and other low- and middle-income countries were limited.

This study observed that the annual number of non-oncological fertility procedures was approximately five times higher than the that of oncofertility. Some patients do not receive counselling because of a lack of communication among their medical teams. In contrast, others are unable to remember the information provided at a time of emotional stress.^(12)^ This study revealed that 60.3% of cancer patients with fertility preservation indicated underwent the procedure. This finding reinforces the importance of specialist consultation to ensure access to and viability of reproductive planning. Fertility preservation improves the subjective experience of patients undergoing cancer treatment, reducing the level of anxiety about the diagnosis and decisional conflicts as well as the chance of delayed decision-making.^(7,13)^ The results of the Preserving Reproductive Opportunity After Cancer Treatment (PROACT) study also showed that patients were satisfied with the information provided and reported that the decision was made after consulting with the fertility specialist.^(7)^

Research on adult female cancer survivors indicated that there are many barriers to fertility preservation.^(6,7,14)^ In our study, the cost, knowledge, acceptance of clinical oncologists, and access to specialized services were considered the main barriers to fertility preservation. In Brazil, the available techniques are expensive and access through the public system is rare and complex. Few services offer assisted reproduction techniques through the public health system. Despite of the urgency for oncologic treatment, the admission process for fertility preservation is often slow. In addition, private health insurance does not cover this process.

In this study, the decision-making process regarding fertility preservation was conducted in a multidisciplinary meeting 70% of the time. Cooperation among specialists is essential to evaluate the best option for patients. Fear of wasting time, worsening oncological prognosis, and the lack of specific protocols for oncofertility were also considered barriers to fertility preservation.^(4,6)^ A retrospective study revealed that among 65 women with various types of cancer, 81.5% were candidates for cryopreservation. Of these candidates, 30.5% refused to undergo embryo, oocyte, or ovarian tissue preservation techniques because of their fear of delayed cancer treatment, worsening prognosis, or lower chance of cure.^(15)^

For many medical oncologists, the lack of training in fertility preservation procedures or knowledge of new available options limits their discussion of oncofertility with patients.^(7)^ More than half of the referrals for oncofertility treatment come from the patients themselves, not from oncologists.^(7)^ This finding suggested that oncofertility is not a routine part of the medical consultation.^(7)^ The American Society of Clinical Oncology (ASCO) recommends that oncology professionals discuss fertility preservation issues and refer patients to a specialist to reinforce and clarify this approach.^(16)^

Almost all the physicians in this study considered oncofertility safe and agreed that it should be indicated for all female cancer patients of reproductive age. This finding reinforces the importance of understanding and applying all aspects of fertility preservation to the decision-making process. The technical procedures for fertility preservation and protocols used for ovarian stimulation at any stages of the cycle or in patients with estrogen-dependent cancer do not seem to interfere with the prognosis or recurrence of the disease.^(17-19)^ In this study, breast and ovarian cancers, leukemia, and lymphoma were the most relevant malignancies for fertility preservation. These findings are in agreement with data from several oncofertility studies.^(6,17,20,21)^

Most infertility experts considered a threshold of 40 years of age for fertility preservation. Other studies suggested an age limit of 43 years.^(7)^ Since it is difficult to determine prospectively whether a patient with a particular cancer type will conceive because fertility can only be verified by the actual birth, advising cancer survivors, who menstruate regularly, on how long they should attempt a spontaneous pregnancy and when they should proceed with assisted reproduction techniques are challenging.^(21)^ There is no agreement on the age limit for the offer of cryopreservation technique. However, age is considered a reliable predictor of successful fertility preservation. In this regard, the cryopreservation of oocytes from individuals over 40 years of age should be considered extraordinarily. Additionally, the low success rates in this situation should be discussed with the patient.^(22)^

The choice of techniques to conserve reproductive function is a non-standard issue, even among specialists. Age, parity, the presence of a partner with whom one wishes to have offspring, the type of cancer, the clinical and social conditions, the gonadotoxicity of the proposed treatment, the time available to perform the proposed fertility preservation technique, the potential of cancer to produce ovarian metastasis, and the inherent risks of hormone therapy or surgical procedure to be performed are all valuable information in this decision-making process.^(3,15,23-27)^

Our study revealed that egg freezing was the technique most frequently used by the respondents. When cryopreservation of embryos is not authorized or refused by patients, cryopreservation of the oocytes is the preferred choice. In this regard, the patient has reproductive autonomy when preserving oocytes rather than embryos as the former provides the cancer survivors with the option to change their partners.^(22)^ Among the available and clinically applicable methods for fertility preservation in young adult women, both embryo and oocyte cryopreservation (slow freezing or vitrification) are the first-line methods. Vitrification of mature oocytes is the preferred technique in post-pubertal women when ovarian stimulation can be controlled and chemotherapy treatment can be delayed.^(16,20,25)^ Embryo freezing requires the patient to undergo an assisted reproduction cycle, with similar limitations to the oocyte freezing process. This technique also assumes that the patient is willing to use a semen donor or has an existing partner who agrees to conceive these embryos.^(20,26)^ Finally, there are numerous ethical and legal issues about the generation and cryopreservation of embryos with no potential use in the future.^(26)^ Ovarian tissue cryopreservation, a technique that ceased to be experimental in 2020, is usually performed by a surgical procedure, which is a limiting factor to be discussed with the patient and his team. In this regard, the efficacy of GNRH agonist and other means of ovarian suppression for oncofertility is still controversial.^(16,25)^

A previous study of cancer patients, who underwent fertility preservation, revealed important differences in the preferred techniques, considering the variables of age, parity, and type of cancer. Women without children preferred more invasive treatments, such as cryopreservation of ovarian tissue (40%) and eggs/embryos (23%) than women with one or more children (19% and 7% for freezing of ovarian tissue and eggs/embryos, respectively). The same study revealed that most women with breast cancer opted for ovarian tissue freezing (45%), followed by egg/embryo freezing (21%). Most lymphoma patients received GNRH agonists (66%), followed by ovarian tissue freezing (33%).^(24)^

Concerning fertility outcomes, most specialists in the present study reported that they failed to follow up patients who underwent oncofertility preservation. The pregnancy and live birth rates were unknown to 50% and 85 % specialists, respectively. This result suggests that most women were possibly cared for in a fragmented way, with no integration among the cancer treatment, assisted reproduction, and obstetrics services. Data on long-term reproductive outcomes are rare in women who received fertility preservation after cancer.^(27,28)^ Medical education and clinical protocols for oncofertility are essential to improve the referral rate and the evaluation of fertility treatments and follow-ups of oncology patients as well as to overcome barriers of service access. Randomized clinical trials of fertility preservation versus non-preservation in women with cancer have indicated that the risk of infertility is not feasible.^(29-33)^

Further research on the pregnancy and live birth rates in women undergoing oncofertility treatment is required. It is essential to know whether reproductive outcomes in these patients are comparable to those in non-oncologic patients and whether there has been a change in the prognosis of the underlying disease in these patients. These data are relevant to support the decision-making process during oncofertility counseling. The results of this study provided important insights about the barriers to improving oncofertility in Brazil. It also allowed us to understand the reasons for the small volume of oncofertility referrals and to understand the specialists’ perspectives on the issue of infertility in cancer patients. This study can help define actions and strategies to effectively integrate fertility preservation into the care model for female cancer patients. All women should have the right to information to make joint decisions with their attending physicians about factors related to oncofertility, such as their clinical condition, age, available time, and costs. The current literature does not authorize us to preserve gametes, which are presumably damaged by antineoplastic treatment, even if the gonadal function is restored. We hope to see the inclusion of oncofertility counseling as a pre-therapeutic step to improve the quality of life in cancer survivors in the near future. Further studies about perceptions of clinical oncologists, hematologists, breast cancer specialists, and female cancer patients on this topic are required in the future.

## Conclusion

This is the first Brazilian study of infertility specialists’ impression about the access to fertility preservation of cancer patients. The study revealed that despite of the recognized relevance of fertility preservation, this procedure is still poorly performed by physicians. This survey had a high response rate and included specialists in all regions of the country. The high proportion of excluded survey results due to incomplete answers may be limitation of this study. However, this issue did not affect the validity of the results. Since this survey focused on infertility specialists in all regions of Brazil, the survey data have good reproducibility and quality. However, most survey participants worked in the private healthcare sector, with only 3% attending the public healthcare system. Additionally, most patients in the public healthcare system do not have access to assisted reproduction services. Therefore, the findings here might only reflect the nature of fertility preservation in the private healthcare system in Brazil.
